# Compact Back-End Electronics with Temperature Compensation and Efficient Data Management for In Situ SiPM-Based Radiation Detection

**DOI:** 10.3390/s23084053

**Published:** 2023-04-17

**Authors:** Nile E. J. Dixon, Stephen D. Monk, James Graham, David Cheneler

**Affiliations:** 1 Engineering Department, Lancaster University, Lancaster LA1 4YW, UK; n.dixon@lancaster.ac.uk (N.E.J.D.); s.monk@lancaster.ac.uk (S.D.M.); 2 Central Laboratory, National Nuclear Laboratory Ltd., Warrington WA3 6AE, UK; james.graham@uknnl.com

**Keywords:** silicon photomultiplier, gamma detectors, temperature compensation, wireless data acquisition systems

## Abstract

A compact back-end interface for silicon photomultipliers (SiPMs) implementing Zener diode-based temperature compensation has been developed for the remote detection of beta and gamma radiation. Remote detection is facilitated by the development of an efficient data management system utilising MySQL database storage for recording periodic spectra data for wireless access over a private Wi-Fi network. A trapezoidal peak shaping algorithm has been implemented on an FPGA for the continuous conversation of pulses from the SiPM, signifying the detection of a radiological particle, into spectra. This system has been designed to fit within a 46 mm cylindrical diameter for in situ characterization, and can be attached to one or more SiPMs used in conjunction with a range of scintillators. LED blink tests have been used to optimise the trapezoidal shaper coefficients to maximise the resolution of the recorded spectra. Experiments with an array of SiPMs integrated with a NaI(Tl) scintillator exposed to sealed sources of Co-60, Cs-137, Na-22 and Am-241 have shown that the detector achieves a peak efficiency of 27.09 ± 0.13% for a gamma peak at 59.54 keV produced by Am-241, and a minimum energy resolution (Delta E/E) of 4.27 ± 1.16% for the 1332.5 keV gamma peak from Co-60.

## 1. Introduction

The detection of beta particles and gamma rays using scintillating materials has become a standard technique. Classically, photomultiplier tubes (PMTs) have been integrated with the scintillators for photon detection, but more recently SiPMs have been a popular alternative despite the effects of temperature-induced drift in the gain given a constant bias voltage [1].

SiPMs are light sensitive devices with many light sensitive microcells consisting of an avalanche diode and quenching resistor. When a photon strikes the diode, it conducts for a short period before being reset. These devices consist of thousands of microcells where multiple microcells can be activated simultaneously, resulting in a current spike proportional to the active number of microcells. SiPMs are preferred over PMTs in some applications, as they are not sensitive to magnetic fields and are compact in size. In addition, SiPMs require lower bias voltages to operate and therefore are more suitable for handheld counting and in situ isotope detection where power infrastructure is limited.

In order to further compact scintillator-based radiation detectors, Field Programmable Gate Arrays (FPGAs) have been integrated into the back-end electronics in order to process pulse data quickly and to interface with peripherals [2,3,4]. Previous work has investigated trapezoidal shaping algorithms that convert detected pulses into alternative forms for better energy resolution, and have been shown to be superior to a quasi-Gaussian shaper [5]. Existing examples of these shapers working on actual hardware [6] use pre-existing FPGA/DAQ boards which limit the detector size and cause a reliance on existing commercial technology.

Storing measured spectra data internally negates the need for an external computer while the detector is running, allowing (along with compacted electronics) for more isolated placement locations where space is a limiting factor. This need is evident in the case of systems such as the PCAMS RadPiper robot [7], a robotic system used to detect U-235 from inside pipe networks; the shape-changing robots PMORPH-1 and PMORPH-2 developed by Hitachi-GE, which can vary their shape to enter nuclear facilities with limited access; and SCORPION, a narrow crawler equipped with a dosimeter for use at the Fukushima Daiichi nuclear power plant [8]. The inspection of small diameter pipes and confined spaces necessitates the use of compact radiation detectors. Another example of the use of compact scintillator-based detectors is for blind-tube monitoring as investigated at Lancaster University [9], where a system consisting of a depth controllable logging probe was used to characterise subsurface radioactivity. Notably, the probe had to fit within a tube with a diameter of 75 mm, and utilised a commercial Topaz-SiPM MCA [10] which was able to meet the size requirements. The Topaz-SiPM MCA cannot store multiple days of spectra data without the use of an external PC/laptop, and cannot natively stream data wirelessly. It is also not clear if the third delay constant (fall time) of the pulse shaper window can be changed, while the device produced here has been tested with a range of pulse shaper window sizes and is able to wirelessly stream the recorded spectra data.

FPGA-based digital pulse processing systems have been implemented previously at The Chengdu University of Technology [6] using COTS data acquisition boards with footprints of 193 mm by 122 mm, and wired USB connections to upload data to a host’s computer. Researchers from Chung-Ang University used a commercial FPGA evaluation board integrated with an ADC board within a PC case [4]; this work had an inherent deadtime where pulses were ignored while the convolution process was unfolding, limiting count rates to approximately 1500 pulses per second. Further examples include the use of a Digitizer XtremeData board containing both FPGA and ADCs [2], and a compact miniature gamma ray spectrometer with a sample rate of 40 MHz and a 12-bit resolution [3]. A paper by Luís Marques [11], published in 2021, compiled commercially available lightweight spectrometers; out of the listed devices, only the Raspix by Crytur has Wi-Fi built in, but this device is unsuitable for use within a 46 mm cylindrical diameter, limiting its possible applications. The listed costs are also far higher than the costs to produce the device proposed here.

Temperature in in situ conditions often cannot be controlled or predicted, with fluctuations caused by time of day, weather, and the heat generated by the surrounding equipment. As temperature affects the breakdown voltage of the SiPM, if the bias voltage is kept constant, the overvoltage will vary, causing a change in gain, in practice spectra recorded over long periods will be skewed, reducing resolution, and features such as photopeaks from gamma emitters could be enshrouded. The impact of temperature fluctuations on energy measurements have also been studied for the MiniPIX TPX3 X-ray detector [12], where it was shown that measured energy peaks were shifted by −27 keV at 81 keV when the detector was heated from 20 °C to 70 °C.

This work investigates a novel temperature stabilisation method to improve the resolution of recorded spectra by reducing the gain deviation of the SiPM(s) caused by the temperatures’ effect on breakdown voltage, resulting in the identification of two suitable electrical components which were implemented when capturing the spectra of four sealed sources.

The work undertaken here also seeks to further compact the required footprint of such a radiation detector, and is intended to be used within confined environments. Other improvements include enhanced data storage and accessibility in order to download the recorded spectrum data, which has been taken over multiple days; this would improve the viability of installing such a detector in remote areas including piping, far above/below ground, and wide area monitoring where the installation of ethernet and other forms of wired communication is not suitable; this could be due to safety concerns with regard to drilling holes for cables in radiologically contaminated environments, or the added trip hazards of adding cables along or across pathways.

## 2. Detector Hardware Design

**Figure 1:**
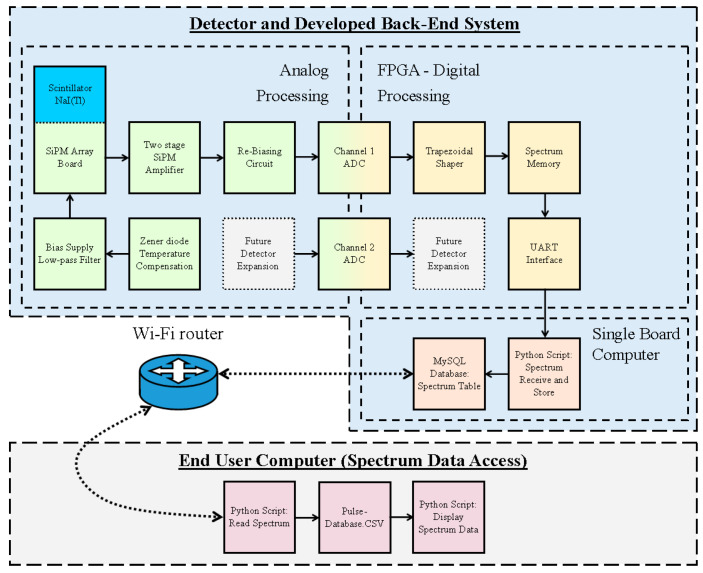
Block diagram of the whole backend system including the digital processing and database access method.

The block diagram depicted in Figure 1 shows the structure of the developed backend system, from the scintillator-based detector through to the method of retrieving data using a Wi-Fi network. Each square represents a discrete part of the system with the following functionality of key areas:

- The Zener Diode Temperature Compensation system has been developed to counter the drift in the gain in the SiPMs due to temperature changes.
- A Bias Supply Low-pass Filter is used to remove high frequency noise from the supply of the SiPM.
- A SiPM Array Board is a circuit board developed to hold a maximum of 17 SiPMs in an array in contact with the scintillator.
- The Two stage SiPM Amplifier converts current pulses from the SiPM into pulses of voltage that can be digitized for further analysis.
- The Re-Biasing Circuit removes dark voltage and adds a set bias to maximise ADC range.
- Channel 1 & 2 ADCs are integrated into a circuit board with the FPGA to digitize pulses from the SiPM.
- The Trapezoidal Shaper is a digital algorithm implemented in VHDL to process pulses from SiPM into a suitable spectrum.
- Spectrum Memory is internal FPGA memory that holds the channel data, recording the amount of interactions detected and their corresponding energy levels.
- Python Script: Spectrum Receive and Store listens for spectrum data collected by the FPGA, formatting it into a MySQL database, while a second script running on a remote PC queries the database to obtain the stored spectrum data.
- The MySQL Database: Spectrum Table database holds every spectrum taken at a set rate (every 1.34 s), allowing a record to be kept and accessed wirelessly at a later point.
- With an End User Computer, an end user can access the database and retrieve the spectra data via a private network and then visualize it using a developed python script.

### 2.1. SiPM Amplifier Design

**Figure 2:**
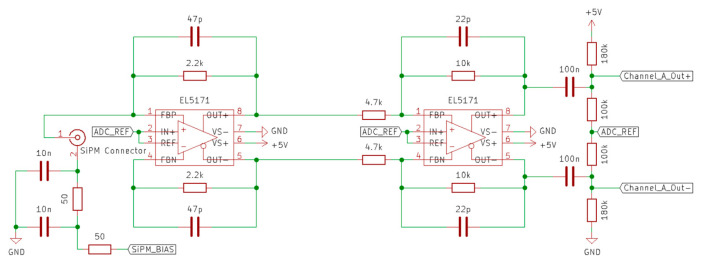
Two stage EL5171 op-amp based amplifier. The first stage is a transimpedance amplifier with differential output, and the second phase is an inverting amplifier, both with a set gain.

Here, SiPM’s (on a C-series semiconductor) [13] have been utilised, which possess a high readout gain of $3\times 10^{6}$. In order to amplify the SiPM output, a transimpedance amplifier is used to convert the produced pulse of current into a measurable voltage pulse that can then be digitised by an ADC for further processing. The amplification board schematic is shown in Figure 2, where the EL5171 op-amp has been implemented, as it can be configured as a single input/dual output transimpedance amplifier.

The two 50 Ω resistors and 10 nF capacitors form a second order low-pass filter in order to reduce noise on the bias supply, which could be further introduced into the amplifier pulse signal. Resistors setting the gain of the first and second stage inverting amplifiers were selected through experimental calibration in order to maximise the ADC range but not result in the clipping of the detected pulses. Capacitors have been added into the feedback of the amplifiers to act as low-pass filters with a frequency cut-off at 723 kHz and 1.54 MHz; as a result, this stretches the pulse, allowing more samples to be taken of its shape by the ADC board, as well as blocking high frequency noise.

**Figure 3:**
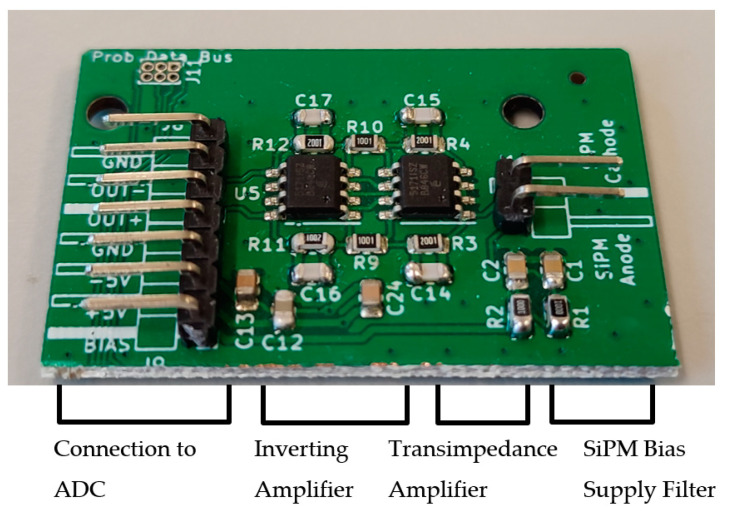
Picture of manufactured SiPM amplifier board labeled with key sections. The board measures approximately 28 by 42 mm.

Between the second stage of amplification and the ADC, a re-biasing circuit has been added, which blocks the SiPM’s dark current, which has been amplified into a voltage. This addition also adds a set DC bias difference of −0.893 V, which allows a greater range of the ADC’s input to be used, which has a peak to peak range of 2 V. It should be noted that the ADC reference voltage has been configured to be 1.5 V on the ADC board. The manufactured SiPM amplifier can be seen in Figure 3.

### 2.2. SiPM Temperature Compensation

To date, multiple methods for the temperature compensation of a SiPM have been investigated and implemented. These methods include the use of a negative temperature coefficient thermistor, serially connected p-n junctions in forward bias [14], and the amplitude of dark pulses from the SiPM [15]. This feedback is then used to adjust the voltage bias across the SiPM in order to keep the overvoltage as constant as possible, and therefore the gain of the SiPM which depends on the overvoltage is stabilized.

Where this work diverges from previous implementations is in the use of Zener diodes as the means of continuously sensing the temperature of the SiPM. These diodes feature a Zener voltage, which is the voltage at which a Zener diode will conduct in reverse bias. However, this is not constant, and instead varies as a function of temperature according to its temperature coefficient, which itself changes from diode to diode due to manufacturing tolerances. The C-series SiPMs possess a breakdown voltage from 24.2 to 24.7 at 21 °C, a recommended overvoltage range from 1.0 to 5.0 V, and a breakdown voltage temperature dependence of 21.5 mV/°C [13]. This allows the breakdown voltage of the SiPM to be represented by:

(1) $$V_{SiPM\;Breakdown}=V_{SiPM-br}+K_{SiPM}(T_{SiPM}-21{}^{\circ}C)$$

(2) $$V_{SiPM\;Breakdown}=24.2+0.0215\times (T_{SiPM}-21{}^{\circ}C)$$

where $V_{SiPM\;Breakdown}$ is the breakdown voltage at a temperature of $T_{SiPM}$ °C, $V_{SiPM-br}$ is the given breakdown voltage at 21 °C, and $K_{SiPM}$ is the temperature dependence of $V_{SiPM\;Breakdown}$. As the SiPM overvoltage is simply the difference between the breakdown voltage and the bias voltage, the overvoltage $V_{SiPM\;Overvoltage}$ can be formulated as:

(3) $$V_{SiPM\;Overvoltage}=V_{SiPM\;Bias}-V_{SiPM-br}-K_{SiPM}(T_{SiPM}-21{}^{\circ}C)$$

**Figure 4:**
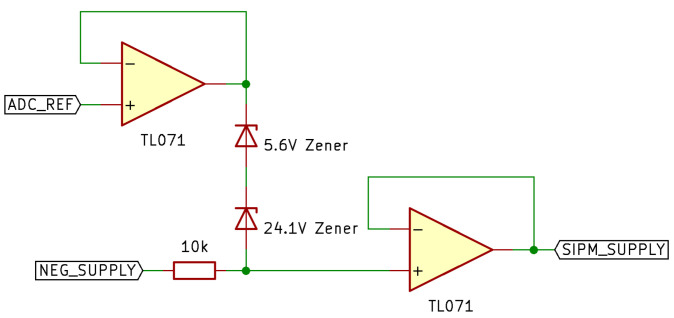
Simplified schematic of simulated temperature compensation circuit.

In order to keep a constant gain, this overvoltage must be kept at a constant value close to the upper end of the overvoltage range in order to maximize the SiPM gain. The breakdown voltage of the Zener diode (Zener Voltage) can also be formulated as:

(4) $$V_{Zener\;Breakdown}=V_{Zener-br}+K_{Zener}(T_{Zener}-21{}^{\circ}C)$$

where $V_{Zener\;Breakdown}$ is the Zener breakdown voltage at a temperature of $T_{Zener}$ °C, $V_{Zener-br}$ is the given breakdown voltage at 21 °C, and $K_{Zener}$ is the temperature dependence of $V_{zener\;br}$. As the voltage drop across the diode is equal to the bias voltage placed over the SiPM (see Figure 4):

(5) $$V_{SiPM\;Bias}=V_{Zener\;Breakdown}$$

(6) $$V_{SiPM\;Overvoltage}=V_{Zener-br}+K_{Zener}\left(T_{Zener}-21{}^{\circ}C\right)-V_{SiPM-br}-K_{SiPM}(T_{SiPM}-21{}^{\circ}C)$$

Therefore, given that the SiPM and Zener diode are held at the same temperature, for the overvoltage to stay constant (perfect temperature matching):

(7) $$V_{Zener-br}=V_{SiPM-br}+V_{SiPM\;Overvoltage}$$

and:

(8) $$K_{Zener}=K_{SiPM}$$

If a suitable Zener diode with the exact required characteristics cannot be located, multiple Zener diodes connected in series can be used instead, as their individual voltage drops and the temperature coefficients would be combined:

(9) $$V_{Zener-1-br}+V_{Zener-2-br}+\ldots =V_{SiPM-br}+V_{SiPM\;Overvoltage}$$

(10) $$K_{Zener-1}+K_{Zener-2}+\ldots =K_{SiPM}$$

### 2.3. Temperature Compensation System Simulation

LTspice (version XVII, Linear Technologies), a SPICE-based analogue electronic circuit simulator, was selected to produce the simulations, as it can utilise models directly from Onsemi, the manufacturer of the Zener diodes, resulting in a more accurate simulation.

In order to simulate the electrical behavior of SiPMs, a simplified Signal Photon Avalanche Diode model (SPAD model) was used. Similar models have been used when focusing on SiPM modeling for front end electronics [16] and SPICE electrical models [17]. This allows the breakdown voltage, terminal capacitance, quench resistance, and diode switching characteristics to also be modeled. For the C-series SiPM, limited model data is provided by the manufacturer, therefore only the terminal capacitance and breakdown voltage were modeled for the SiPM in this work.

The SiPM has been implemented in the simulation using three components: a voltage source, which varies using Equation (2), a digital switch with a short on duration which represents a single photon striking the SiPM, and a 3400 pF capacitor representing the total capacitance across the anode and cathode of the SiPM.

Figure 4 shows the temperature compensation system using a combination of two Zener diodes in series. These diodes have Zener voltages of 5.6 V and 24.1 V, respectively, making the total series voltage drop by 29.7 V. This circuit has been simulated in LTspice alongside a non-temperature controlled ‘static’ system using a set bias voltage created from a voltage divider. The bias voltage of both the temperature compensated and non-temperature compensated systems have been made to match at a temperature of 27 °C. The two Zener diodes have been configured in reverse voltage bias, with two unity gain amplifiers added in order to, firstly, isolate both the reference voltage (ADC_REF) from the cathode of the upper Zener, and secondly to act as a current buffer for the Zener voltage drop in order to bias one or multiple SiPMs. The TL071 op-amp was used for the buffer op-amps.

**Figure 5:**
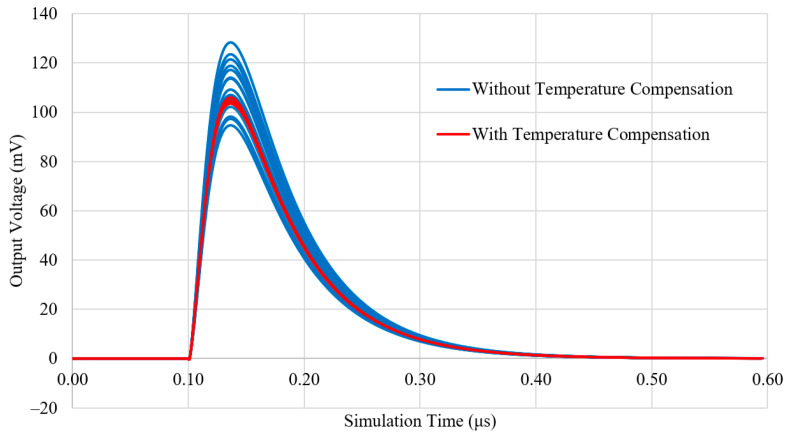
LTspice simulation of an amplified pulse from the two systems over the temperature range of −20 to 50 °C with a step of 5 °C. Traces of blue and red correspond to the non-compensated and Zener compensated systems, respectively.

**Figure 6:**
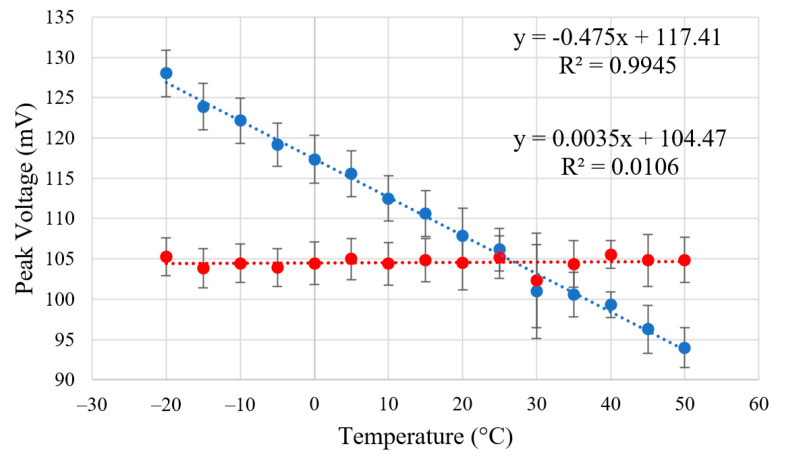
LTspice simulation plotting the peak to peak magnitude of each simulated pulse against temperature °C. Blue and red points correspond to the non-compensated and Zener compensated system, respectively, both fitted with a linear dotted line. Three sigma error bars have been added based on the results of 10 simulations.

As a result of the LTspice simulations with the temperature compensated and non-temperature compensated systems, Figure 5 and Figure 6 have been obtained. These show the resulting pulses from the output of the transimpedance amplifier and their peak-to-peak measurements over the temperature range of −20 to 50 °C with a step of 5 °C. Without compensation, the slope of best fit has a gradient of −0.475 ± 0.020 and an intercept of 117.41 ± 0.51, compared to the compensated system with a fit gradient of 0.0035 ± 0.019 and an intercept of 104.47 ± 0.49 (a 95% confidence interval). Therefore, it has been shown that the simulated drift in pulse magnitude has been reduced by more than a factor using the Zener compensation method over the tested temperature range.

#### Zener Diode Selection

**Figure 7:**
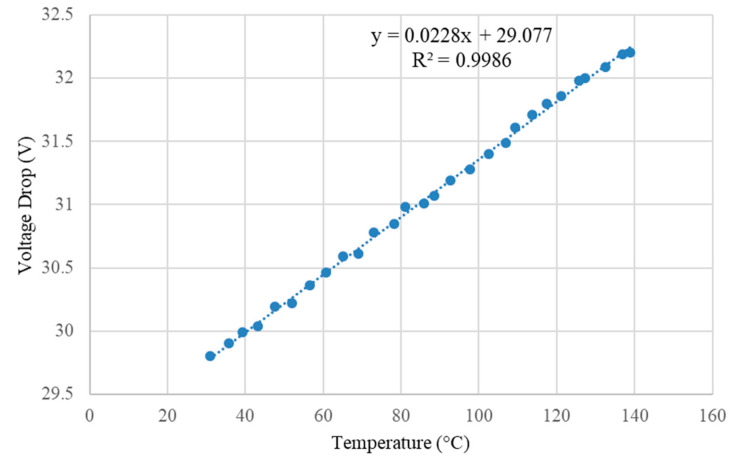
Measured voltage drop of the combined Zener diodes over the temperature range of 30 to 140 °C. A negative supply voltage of 32.61 V and a 10 kΩ biasing resistor was used. A linear line of best fit (dotted blue line) has been added to estimate the temperature dependance and voltage drop of the combined Zener diodes at 0 °C. Voltages were measured to ±0.01 V accuracy, and temperature was measured to ±1 °C accuracy.

While the simulations performed in the previous section deal with Zener diodes with set temperature coefficients, in practice, the diodes have a range of values in which the coefficients can lie between, as defined by the manufacturer. Therefore, an experiment was conducted in order to find two suitable Zener diodes that match the SiPM. The experiment involved using a temperature controllable hotplate (Voltera V-One) on which the Zener diodes were attached. An oscilloscope was used to measure the voltage drop over the two Zener diodes, and an infrared thermometer was used to measure the surface temperature of the Zener diodes. The relationship between temperature and voltage drop for the selected diodes is shown in Figure 7.

**Figure 8:**
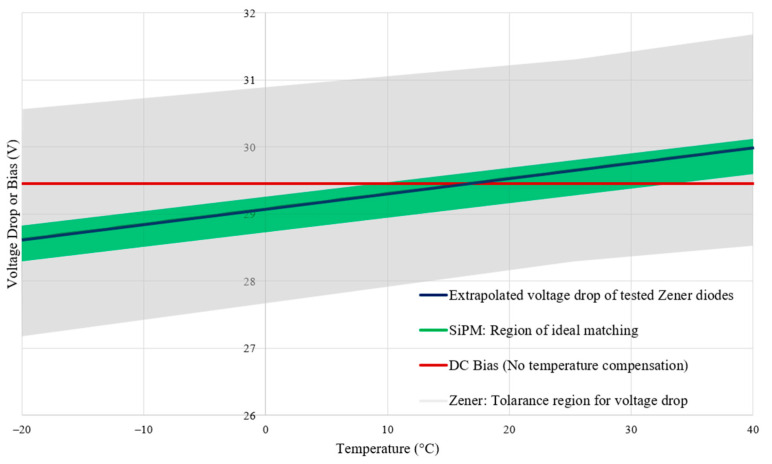
Combined plot of the extrapolated voltage drop caused by the measured Zener diodes compared with a set DC bias, ideal voltage drop region for biasing the SiPM, and the region of tolerance given to the Zener diode’s voltage drop by the manufacturer.

By fitting a linear trend line, the temperature coefficient can be shown to be 22.8 mV/°C with a voltage drop of 29.56 V at 21 °C. This trend line can be extrapolated for a more suitable temperature range (selected to be −20 to 30 °C), as seen in Figure 8.

In Figure 8, additional data has been plotted alongside the extrapolation of the measured Zener diode temperature drop (represented as a blue line). A red line represents a compresence to a set bias voltage (29.45 V) which is constant, unaffected by temperature. The green shaded region encapsulates the ideal bias voltage of the C-series SiPM for an overvoltage of 5 V above the breakdown voltage, and the exact breakdown voltage varies from part to part due to manufacturing tolerances [13]. Finally, a gray shaded region represents the tolerances in which the combined Zener pair’s voltage drop could lie; these tolerances have been taken from the respective datasheets of the diodes (see Table 1).

Interpreting Figure 8, using the set DC bias at 29.45 V would not be suitable below a temperature of approximately 10 °C, as the voltage drop leaves the SiPM’s region of ideal matching, resulting in an overvoltage greater than 5 V, which could cause permanent damage to the SiPM, while the Zener voltage drop stays within the ideal region for the full temperature range. Table 1 states the device specifications taken from the respective datasheets.

As the measured Zener diodes have a 22.8 mV/°C temperature coefficient and the SiPM has a defined 21.5 mV/°C temperature coefficient, the resulting bias error for the tested Zener diodes with a C-series SiPM can be defined using the measured Zener diode behaviour and Equation (6):

(11) $$V_{Overvoltage\;error}=0.11+0.0013T\;{}^{\circ}C\pm 0.25$$

where $V_{Overvoltage\;error}$ is the difference in bias voltage caused by the imperfect matching when the SiPM(s) and Zener diodes are both held at a temperature *T* °C. The ±0.25 represents the SiPMs breakdown voltage tolerance. Therefore, the implementation of this tested system would reduce the temperature dependence of the SiPM overvoltage from 21.5 mV/°C to 1.3 mV/°C.

### 2.4. FPGA and ADC Interconnection Board

In order to digitize the pulses from the SiPM amplification board, a digitiser board utilising a 14-Bit analog to digital converter (AD9244 [20]) was used. The ADC interconnection board was designed, manufactured and integrated into the detector to run at a sample rate of 50 MSPS. Two ADCs were included, allowing for the possibility of two separate channels operating simultaneously, providing an improvement over systems such as the Topaz-SiPM, which only has one input channel without an option to add an additional channel. Compared with another similar FPGA-based wireless gamma spectrometer [3], the system developed here has a higher resolution ADC, whilst also having a greater sample rate.

**Figure 9:**
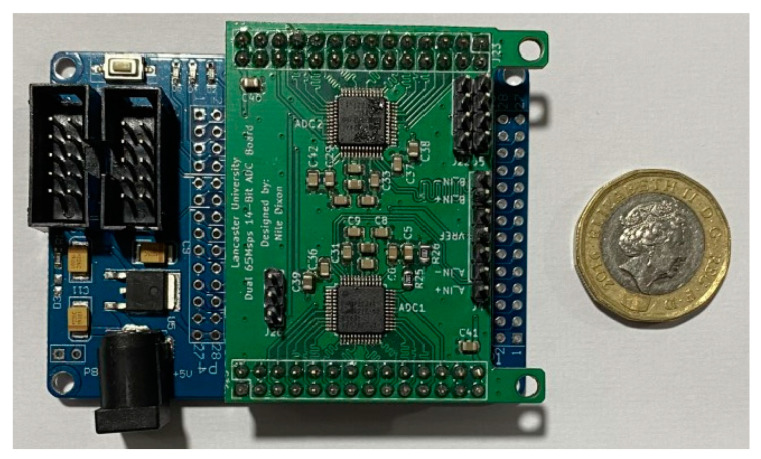
Image of a manufactured ADC board, populated with two AD9244 ADCs and supporting circuitry, sitting on top of the Cyclone II minimal development board. A pound coin is shown for scale.

An EP2C5T144 Altera Cyclone II FPGA soldered onto a minimal development board was used for running the ADC communication, serial communication, and pulse shaping algorithms. The Cyclone II was selected due to its compact size and large I/O pinout. The ADC board fits atop of the FPGA minimal development board to be as compact as possible and has added connections for +5 V, +3.3 V, ground, input pairs for each ADC, a voltage reference output, and a UART interface for sending data to the single board computer, a Raspberry Pi Zero 2 W. A photograph of the dual ADC board attached to the FPGA board can be seen below in Figure 9.

To further compact the FPGA to match the footprint of the ADC board, the JTAG, active serial header, and regulator were removed. The 1.2v regulator used to supply the FPGA was migrated onto the new cutdown board and soldered alongside new pull up resistors for the programming interfaces, without which the FPGA will not start. As a result, the original FPGA board footprint was reduced from 72 mm by 50 mm to 39 mm by 50 mm, which is identical to the ADC board. While powered, the FPGA and two ADCs pull approximately 220 mA, with a further 260 mA used by the single board computer, and less than 1 μA is used by the SiPM bias. For communication between the FPGA and ADCs, two headers provide two 14-bit parallel data buses and connections for the clock 50% duty cycle stabilizer, the out-of-range indicator, and data format pins for configurability.

### 2.5. Digital Signal Processing

There are multiple methods of detecting and measuring the characteristics of pulses emitted by an amplified SiPM. Past research has investigated the use of trapezoidal and triangular pulse shaper algorithms that take the digitized samples of exponentially decaying pulses and convert them into another form that can be more easily analyzed [5]. Multiple shapers have also been implemented with different “fast” and “slow” shaping constants so that multiple pulses that overlap (double counts) can be identified and removed [6].

Here, a trapezoidal shaping algorithm has been implemented [5] in VHDL within the Cyclone II FPGA and AD9244 ADC in order to create and store energy spectra formed from the pulses emitted by the SiPM. The key variables that affect the output of the shaper are three sequential sample delays that will be referred to as simply delay 1, 2, and 3. Also present is a threshold value that dictates when a pulse is present above a background of noise, and finally an M coefficient that is used for zero pole cancellation, which depends on the exponential decay time of the SiPM pulses and the sample rate of the ADC [21].

#### FPGA Spectrum Accumulation

In this mode of operation, a two port RAM module is implemented on the FPGA, which allows for both the writing and reading of 16-bit values simultaneously at two different clock frequencies. When a pulse is emitted by the SiPM, it is amplified, digitized, and shaped; the peak value of the shaped pulse is then used as the address for recording that interaction. Proceeding a set delay after the pulse threshold trigger, the current count stored in the address of the peak value is read. On the next clock cycle, the read count value is incremented by one and then written at the same memory address, thereby representing a recorded interaction at that energy.

**Figure 10:**
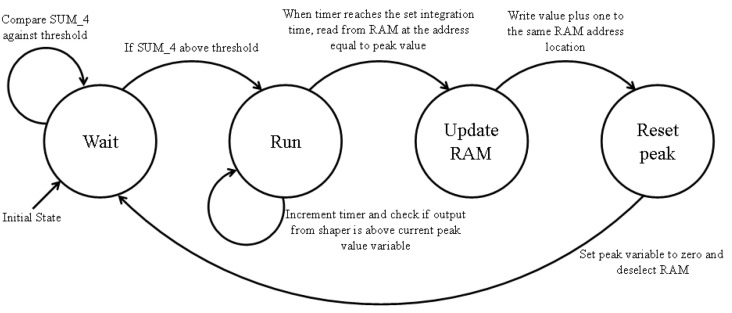
Finite state machine of the pulse peak storage system implemented on Cyclone II FPGA. Each transition occurs every clock cycle. The variable SUM_4 is the output of the shaper algorithm.

Figure 10 shows a finite state machine of this memory writing process. The output from the shaper algorithm produces a 32-bit integer which is divided down to 16 bits for the detected peaks to fit within the limited spectrum memory on the FPGA. This also allows the energy range to be adjusted to suit the energies of the different radiation sources the detector will be tested with, as the detector energy channels scale proportionally to the division factor. From here onwards, the factor division of $2^{18}$ and $2^{17}$ will be referred to as zoom level 1 and zoom level 0.5, respectively. This VHDL code was simulated using Modelsim-Altera [22] before running on the detector hardware.

### 2.6. Database Design and Implementation

As this detector is primarily designed for portability and in-situ use with minimal infrastructure, there is a need to provide a method of onboard data storage, as the end user may not always be available to retrieve data captured by the detector in real time. A MySQL database was implemented in the detector to store the spectra data as it is streamed from the FPGA. MySQL is a well-suited internet of things solution, as multiple copies of this detector can be connected to a single network, each with a unique IP address allowing access through a secure SSH connection. The FPGA talks to the single board computer hosting the database using UART at a baud rate of 115,200 symbols per second. An exposure of 1.34 s was used for collecting each spectrum before the FPGA delivers the contents of the channel memory, and while doing so it resets every channel to zero, preparing for the next exposure. A python script listens for this data, where it is then formatted and inserted as a new entry into the database as a BLOB (Binary Large Object) alongside the time and date. When the user wishes to receive data wirelessly from the detector, the destination computer is connected to the same private network as the detector, and a second python script on the destination computer queries each entry of the database, removing it afterwards. The developed database has been tested up to a maximum table size of 161,717 rows of spectra data stored internally on the detector. The total count time for this data can be calculated using:

(12) Count Time = Database Rows × Exposure Time

(13) Total Count Time = 161,717 × 1.34 S = 216,700 S = 2.51 Days

## 3. Detector Resolution Determination Using LED Pulse Testing

Here, the resolution of the captured spectra was benchmarked using Light Emitting Diode (LED) pulse testing. This involves placing a LED in a set position above the SiPM, where it is fed voltage pulses to modulate the LED and thus test the efficacy of the SiPM detecting the bursts of light. The blinker circuit produces a constant pulse of light with a period of approximately 304 ns. The frequency of the blinking and the LED brightness were kept constant for all testing, in order to study the effects on the recorded spectra caused by adding SiPMs and changing the delay constants of the pulse shaper. The LED was kept at a constant distance of approximately 5 cm from the SiPM within a lightproof enclosure for the three experiments.

**Figure 11:**
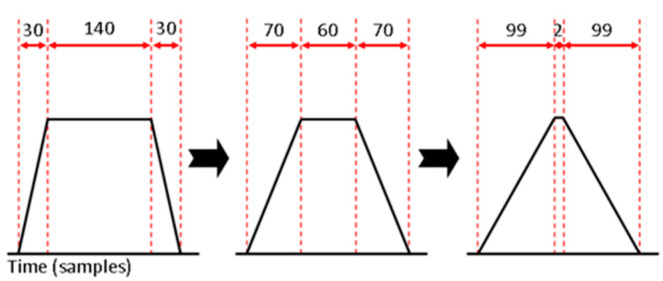
Visual representation of the effect of the delay values on the shape of the window, i.e., for the far-left example: delay 1, 3 = 30 samples, delay 2 = 140 samples.

The delays of the implemented shaper have been selected in order to transition from a trapezoidal to a triangular shaped window to see the effect on the calculated resolution. Figure 11 shows three examples of the transition from a trapezoidal to a triangular shaper. For the experiment, seven different windows were used in the transition, and the sum of the three delays has been kept constant at 200 samples in order to study the shaper effect on the resulting spectra.

### 3.1. LED Blinking Experiment Results

**Figure 12:**
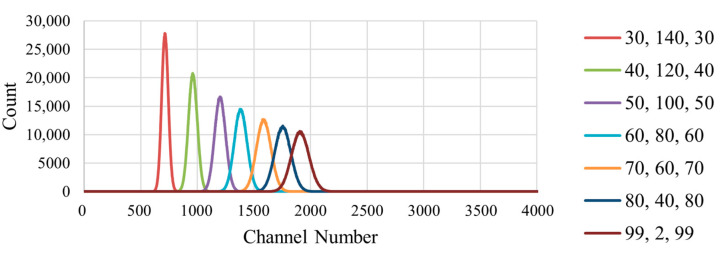
Combined raw unfiltered spectra of 8 LED blink tests with a single SiPM. The legend indicates the duration of delays 1, 2, and 3, respectively.

The recorded spectrum for each window with 1, 2 and 3 SiPMs was taken over 13.4 min made from a cumulative total of 600 individual spectra taken every 1.34 s. The FWHM and resolution were obtained from the recording by placing a Gaussian fit over the peak. The recorded set of spectra for one SiPM has been plotted in Figure 12 as an example, but the data has also been produced for tests with 2 and 3 SiPMs. The graph legend has been formatted with the values selected for delay 1, delay 2, and delay 3, respectively. The detector resolution has also been calculated using Equation (14), as it is an indicator of how capable the detector is in terms of distinguishing energy.

(14) $$Resolution=\frac{FWHM}{Peak\;Channel}\times 100\%$$

As expected, a single peak is captured individually for each test run with a different shaping window. The peaks are at increasingly higher channels when produced by windows with decreasing delay 2 values, i.e., as the window becomes more triangular. This trend continued when using 1, 2 or 3 SiPMs. Comparing the number of SiPMs used, a positive shift in the detected peak was seen with each additional SiPM, as increasing the detectors’ sensitive area has increased the voltage magnitude of the output pulses, as it can collect more light from the LED.

The ratios between the number of SiPMs are important in gauging the benefit of having multiples of them. In an ideal case, doubling the sensitive area of the detector would result in a peak ratio of 2, as the pulse would be twice the magnitude, as the two spikes of current from each SiPM would sum together. By taking the ratio between the peak channels for one and two SiPMs (Delays: 99, 2, 99), the gain is found as 1.60, and between one and three SiPMs (Delays: 99, 2, 99) as 1.88. One contribution for this gain inefficiency is the combined capacitance between the anode and cathode of each SiPM affecting the rise time of the pulse. This can be improved using active summation [23], although at an added cost and complexity.

**Figure 13:**
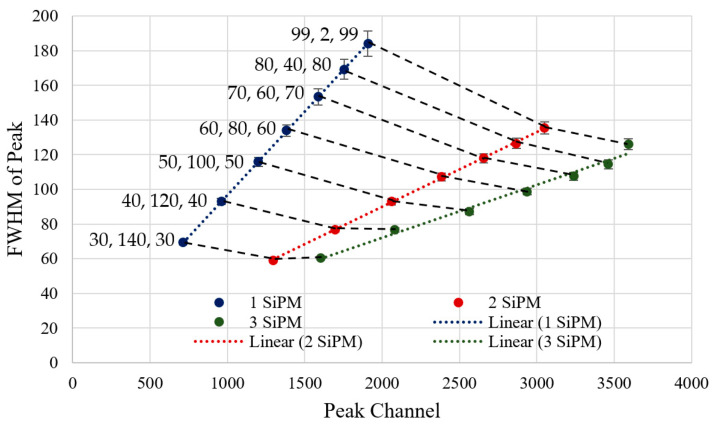
Graph of channel FWHM from each detected peak across all LED testing experiments, with three-sigma error bars. Three lines of best fit consisting of coloured dotted lines have been added on each set of results for 1, 2, and 3 SiPMs, respectively.

**Figure 14:**
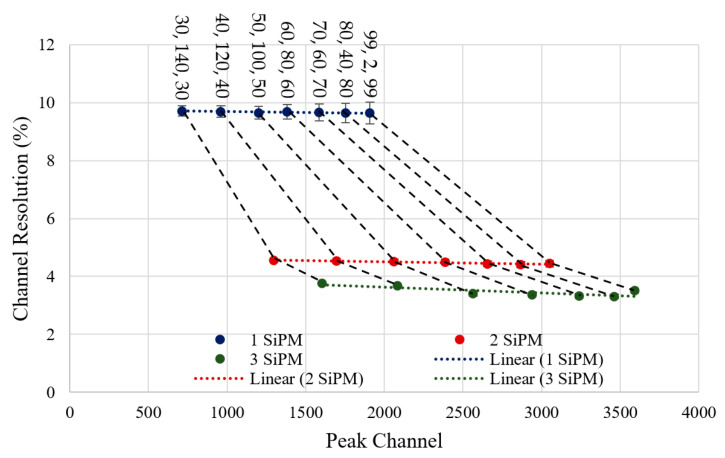
Graph of channel resolution from each detected peak across all LED testing experiments, with three-sigma error bars. Three lines of best fit consisting of coloured dotted lines have been added on each set of resolution values for 1, 2, and 3 SiPMs, respectively.

The channel FWHM and resolution for all LED experiments are plotted in Figure 13 and Figure 14 with a linear fit added for each SiPM count. Black dotted lines have also been added to link the results using the same shaper delays. Interpreting Figure 13, the FWHM and peak channel tends to increase as the shaper becomes more triangular. While an increased FWHM is not advantageous, when combined with the apparent gain increase, the resolution (Figure 14) tends to stay relatively constant for all tested shaper shapes. With the change of one to two SiPMs improving the resolution by an average factor of 2.16, and the change from one to three SiPMs improving by an average factor of 2.79.

### 3.2. Temperature Compensation Experiment Results

**Figure 15:**
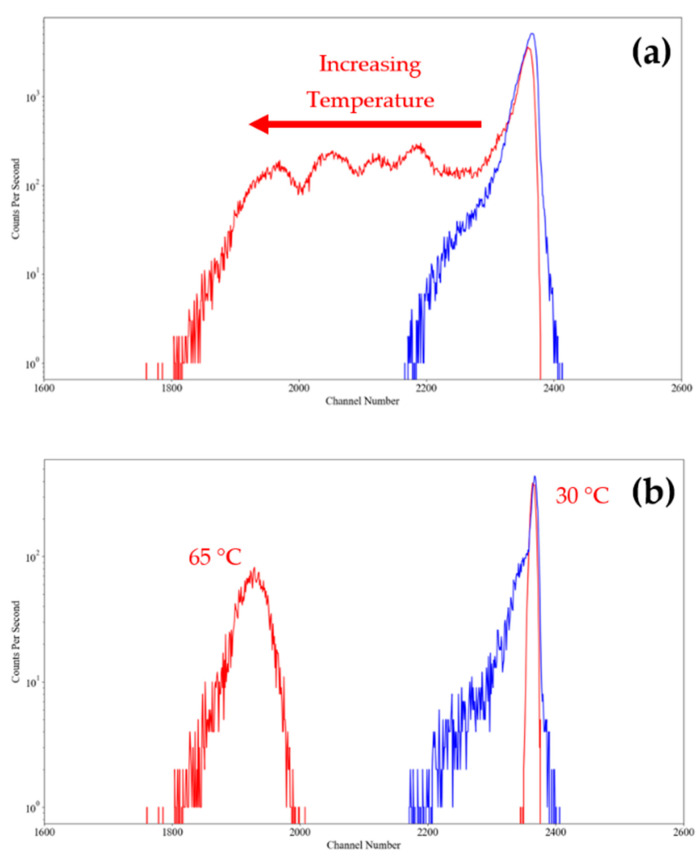
A log graph (**a**) plotting cumlative spectra taken over the full heating experiment. A log graph (**b**) of individual spectra taken at 30 °C and 65 °C, respectively, for both compensated (blue trace) and non-compensated (red trace) systems. Only channels 1600 to 2600 have been displayed for purposes of clarity.

This experiment has been used to validate a Zener temperature compensation system; the SiPM and Zener diodes were placed onto a high-power resistor, the power resistor was used to gradually heat the SiPM from a starting temperature of 30 °C to 65 °C, while the detector was collecting the spectra of a constant brightness and constant blinking LED. A slightly lower Zener voltage was used (3.9 V replacing 5.6 V) to ensure that the overvoltage stayed below 5 V. The resulting data has been plotted in Figure 15.

As expected, in Figure 15 a clear shift can be seen in the non-compensated system when comparing its spectra taken at 30 °C to its spectra taken at 65 °C; this is explained as the overvoltage decreases as the SiPM’s temperature increases, therefore lowering the gain of the system at higher temperatures. The temperature controlled system has undergone a small shift resulting in a side tail; this could be due to noise added by the increase in dark counts or the non-ideal Zener diode matching, but has remained as a single peak over the test duration.

## 4. Sealed Source Calibration with Temperature Compensation

The purpose of these experiments is to calibrate and validate the backend system to show that it is capable of radiation detection. The experimental set-up is as follows: the window of a cylindrical (40 mm dia. by 52 mm long) Na(Tl) scintillator manufactured by advatech [24] was placed onto the SiPM board containing three C-series SiPMs, using optical grade silicone grease to improve the light transfer between them. Along with the SiPMs, the amplifier and temperature compensation boards were placed together inside a lightproof enclosure. Sealed point sources Co-60, Cs-137, Na-22, and Am-241 (with the respective activities of 4.46, 27.27, 0.60, and 33.51 kBq) were individually placed directly upon the cylindrical scintillator before the enclosure was closed.

**Figure 16:**
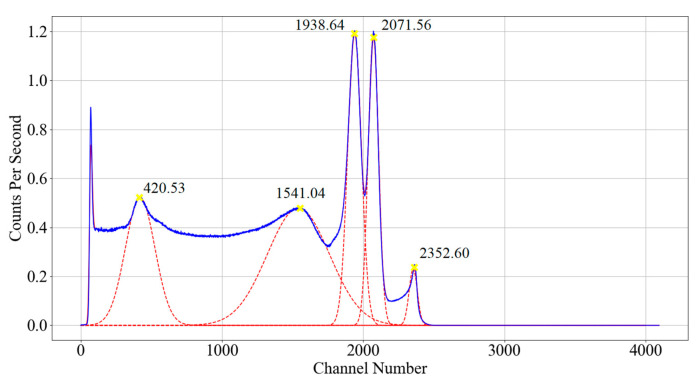
The spectrum of theCo-60 sealed source with the background removed taken at zoom level 1, blue trace: raw channel data, solid red trace: 20 sample moving average, dashed red trace: Gaussian fit around peaks (capture time: 19.76 h, for an average of 1002.85 CPS).

Figure 16 shows the spectra recorded by the developed detector for a sealed source of Co-60; this experiment was repeated for sources Na-22, Cs-137 and Am-241, respectively. Two background spectra at zoom levels 1 and 0.5 were recorded before testing, and have been subtracted from the captured spectra of each source. Finally, a Gaussian fit was applied to each detected peak using 50 channels below and above the peak channel, and is displayed in Figure 16 as a dashed red line. The point at which the ADC saturates can be seen as a peak close to channel 2352.60 ± 4.21. Any pulses that saturate the ADC congregate together into this peak distribution, and as such will be excluded from the peak analysis.

**Figure 17:**
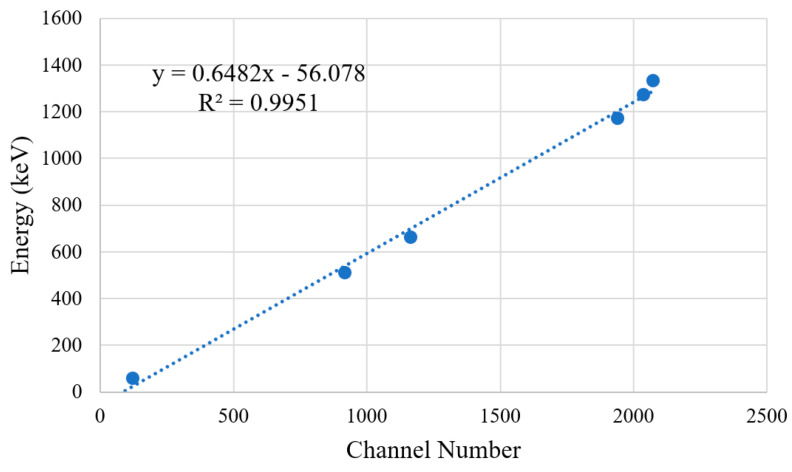
Graph of the calibration fit for the detector with three-sigma error bars based upon the collected peak channel data in Table 2. A linear line of best fit (dotted blue line) has been fitted to the data in order to relate each channel to a detection energy. The fit has a gradient and intercept standard error of 0.65 ± 0.02 and −56.08 ± 35.39, respectively (a 95% confidence interval).

**Figure 18:**
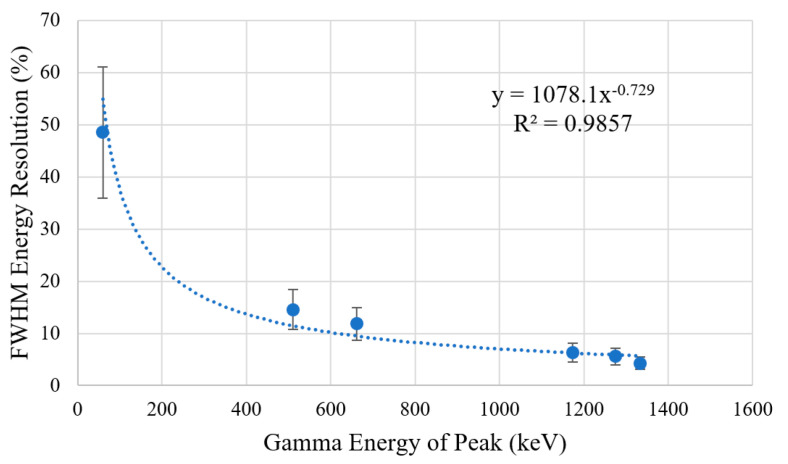
Graph of the energy resolution of the detector with three-sigma error bars. A power trend line has been added to the data alongside a $R^{2}$ value for the fit.

Table 2 displays the combination of detected peaks in each recorded spectrum with a known energy expected for the tested radioisotope. This was then used to plot a calibration fit for the detector (Figure 17). The lowest peak from Am-241 was not used due to the uncertainty in the recorded counts caused by the overlap with X-rays from its daughter nuclide Np-237. The resolution for each photopeak has been calculated and plotted in Figure 18 by dividing the FWHM by the assigned photopeak energy.

From Figure 17, a clear correlation between channel number and energy can be identified with a linear fit and an $R^{2}$ value of 0.9951. From this, the detector calibration Equation (15) is obtained.

(15) $$Channel\;Energy\;\left(keV\right)\;=\;0.6482\;\times \;Channel\;Number\;-\;56.078$$

Therefore, each channel represents 0.6482 keV at zoom level 1 and 0.3241 keV at zoom level 0.5. The saturation peak in Figure 16 can be used with Equation (15), indicating that the detector begins to saturate approaching an energy of 1468.88 ± 2.73 keV.

**Figure 19:**
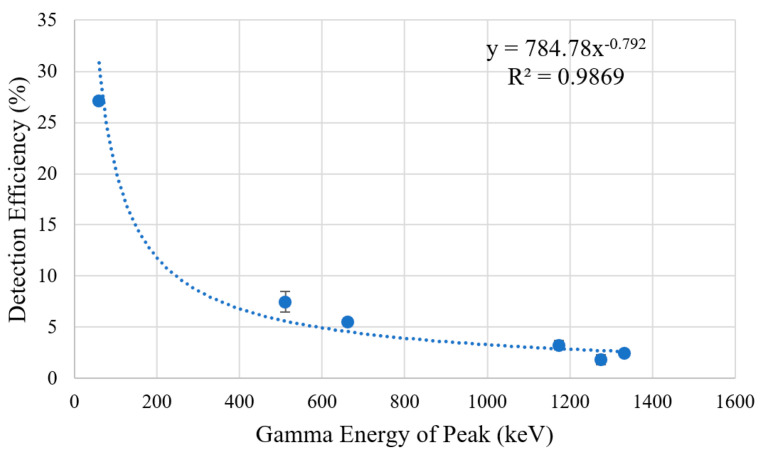
Plot of the calculated detection efficiency for each peak in the recorded spectras with three-sigma error bars. A power trend line has been added to the data alongside the $R^{2}$ value for the fit.

The calculated FWHM energy for each Gaussian fit peak is displayed in Table 2 and plotted in Figure 18, followed by the detection efficiencies of each Gaussian fit peak displayed in Table 3 and plotted in Figure 19. The results show that the FWHM energy resolution and efficiency increases as the energy of the detected gamma peak decreases. This relationship can also be seen in other publications working with similar systems [3,28,30]. The detector achieves a peak efficiency of 27.09 ± 0.13% for a gamma peak at 59.54 keV produced by Am-241.

## 5. Conclusions

The work presented in this paper shows the development of backend electronics capable of amplifying, digitizing, and shaping SiPM pulses for scintillator-based gamma spectroscopy. This has been validated and calibrated experimentally using LED blink testing and exposure to radiation using sealed sources of Co-60, Cs-137, Na-22 and Am-24. Furthermore, a MySQL database system has been developed to store the spectra data recorded in the experiments, each having a total of 4096 active channels. The database has been successfully tested with up to 161,717 entries, showing that the detector can be left to record spectra unsupervised for multiple days. By recording spectra at regular intervals, this allows the future analysis of the recorded data to not only find which radioisotopes were detected, but also over what period of time, which is ideal for transient surveying applications.

Circuits have been produced, including a SiPM amplifier and a dual ADC board capable of digitizing SiPM pulses for trapezoid shaping. These boards, along with a single board computer, can fit within a cylindrical diameter of 46 mm. The fully produced detector system is also low cost, with the majority cost being that of the scintillator. A VHDL implementation of a trapezoidal shaper for the processing of SiPM pulses into a spectrum has also been produced and validated experimentally for different numbers of SiPMs. This showed that using multiple SiPMs in parallel improved the resolution of the peaks in the detected spectra, while the shaper window did not have a large effect on the resolution. On the other hand, the shaping window affected the gain of the system, finding that a triangular window resulted in peaks at higher channel numbers compared to trapezial windows for a set total delay length.

The sealed source radiation testing of the backend electrical system with a Na(Tl) scintillator has produced a range of spectra used in obtaining the resolution and detection efficiency of the detector over the range of detected energies. We found a minimum energy resolution (Delta E/E) of 4.27 ± 1.16% for the 1332.5 keV gamma peak from Co-60 and a maximum detection efficiency of 27.09 ± 0.13% from a 59.54 keV Am-241 gamma peak. A fit has been produced to relate the channel number to energy, finding that the detector has a detectable energy range from 56.08 keV to 1468.88 ± 2.73 keV, and a resolution of 648 eV at zoom level 1.

Finally, a new temperature compensation system has been designed and simulated using two serially connected Zener diodes. Temperature experiments have also found two suitable components, that being a SZMM5Z24VT1G and a SZMM3Z5V6ST1G Zener diode in combination, which would reduce the temperature dependence of the SiPM’s overvoltage from 21.5 mV/°C to 1.3 mV/°C. The Zener temperature compensation system was also validated experimentally using a heating experiment, showing a lower shift in detected peaks within the recorded spectra when compared to a set bias system, over a temperature range of 30 to 65 °C.

## Figures and Tables

**Table 1:** Tolerances for voltage drop and temperature coefficients [13,18,19].

Specification	SiPM (with 5 V Overvoltage)	Zener Diode 5.6 V	Zener Diode 24.2 V	Zener Diodes Combined
Min Voltage Drop (V)	29.2	5.49	22.8	28.29
Max Voltage Drop (V)	29.7	5.73	25.6	31.33
Min Temperature coefficient (mV/°C)	21.5	−2	18.4	16.4
Max Temperature coefficient (mV/°C)	21.5	2.5	22	24.5

**Table 2:** Calibration table of peak channel detected for photopeak, backscatter and Compton edge of the tested sealed sources of Co-60 [25], Cs-137 [26], Na-22 [27,28] and Am-241 [29].

Isotope	CPS	Collection Time (h)	Feature	Peak Channel (3σ)	Energy (keV)	Resolution (%) (3σ)	FWHM (keV) (3σ)
Co-60	1002.85	19.76	Photopeak	1938.64 ± 3.35	1173.24	6.30 ± 1.82	73.89 ± 21.30
			Photopeak	2071.56 ± 2.23	1332.50	4.27 ± 1.16	56.86 ± 15.47
			Compton edge	1541.04 ± 132.30	-	-	-
			Backscatter	420.53 ± 21.70	-	-	-
Cs-137	3614.40	9.45	Photopeak	1164.38 ± 0.78	661.66	11.82 ± 3.07	78.18 ± 20.29
			Backscatter	371.88 ± 6.26	-	-	-
Na-22	237.53	9.90	Photopeak	918.13 ± 2.39	511.00	14.52 ± 3.82	74.19 ± 19.53
			Photopeak	2035.70 ± 4.88	1274.53	5.57 ± 1.55	71.01 ± 19.78
Am-241	3572.21	12.82	Photopeak	123.60 ± 0.19	59.54	48.54 ± 12.58	28.90 ± 7.49
			Photopeak	71.53 ± 3.15	26.34	-	-

**Table 3:** Detection efficiency of each gamma peak, with all peak channels adjusted to a zoom of 1.

Specification	Fitted Gamma Energy (keV)	Decay Branching Ratio	Detected Count Rate (CPS) (3σ)	Detection Efficiency (%) (3σ)
Co-60	1173.24	1.00	144.79 ± 20.00	3.25 ± 0.45
	1332.50	1.00	110.18 ± 11.15	2.47 ± 0.25
Cs-137	661.66	0.85	1269.48 ± 30.90	5.48 ± 0.11
Na-22	511.00	1.81	81.50 ± 6.08	7.48 ± 1.01
	1274.53	1.00	17.06 ± 2.80	1.84 ± 0.47
Am-241	59.54	0.36 [29]	3268.31 ± 45.20	27.09 ± 0.13

## Data Availability

The data presented in this work are available from the corresponding author.
